# Exopolysaccharide (EPS)-Producing *Streptococcus thermophilus*: Functional and Probiotic Potential

**DOI:** 10.3390/foods14173013

**Published:** 2025-08-28

**Authors:** Dominika Jurášková, Vanessa C. Pires, Susana C. Ribeiro, Sónia S. Ferreira, Fábio Bernardo, Dmitry Evtyugin, Manuel A. Coimbra, Celia C. G. Silva

**Affiliations:** 1 Institute of Agricultural and Environmental Research and Technology (IITAA), University of the Azores, 9700-042 Angra do Heroísmo, Portugal; dominika.juraskova@gmail.com (D.J.); vanessacorvelopires@gmail.com (V.C.P.); susana.ic.ribeiro@uac.pt (S.C.R.); 2 LAQV-REQUIMTE, Department of Chemistry, University of Aveiro, 3810-193 Aveiro, Portugal; soniasferreira@ua.pt (S.S.F.); mac@ua.pt (M.A.C.); 3 CICECO—Aveiro Institute of Materials, Department of Chemistry, University of Aveiro, 3810-193 Aveiro, Portugal; fabiobernardo@ua.pt (F.B.); dmitrye@ua.pt (D.E.)

**Keywords:** *S. thermophilus*, exopolysaccharides, lactic acid bacteria, probiotics, EPS

## Abstract

This study presents a characterization of exopolysaccharide (EPS)-producing *Streptococcus thermophilus* strains isolated from goat milk, including information about structural and functional characteristics of EPS. The isolates exhibited efficient lactose fermentation, broad carbohydrate utilization, and desirable enzymatic activities for technological applications, particularly aminopeptidases and acid phosphatase, while lacking harmful enzymes and virulence traits. Among the four strains studied, GM4 emerged as a particularly promising probiotic due to its sensitivity to all tested antibiotics, high β-galactosidase activity (56.2 × 10^3^ Miller units), moderate antioxidant capacity (scavenging 22.7% of DPPH and 5.7% hydroxyl radicals), cholesterol-lowering ability (26.9%), high auto-aggregation capacity (46.8%), and co-aggregation (>30%) with key foodborne pathogens including *Escherichia coli*, *Listeria monocytogenes*, and *Staphylococcus aureus*. GM4 produced an EPS with high dextranase resistance, and its production was enhanced in lactose-enriched media (yield = 2.58 g/L). The purified EPS consisted of two heteropolysaccharides (12.0 kDa and 112 kDa), primarily composed of glucose (Glc, 53%) and mannose (Man, 29%), with minor contributions from uronic acids (8%), ribose (6%), arabinose (2%), and galactose (2%). Key glycosidic linkages included (1→4)-Glc, (1→2,6)-Man, (1→2)-Man, and (1→4,6)-Glc. Functional assays demonstrated notable antioxidant activity, with 52.5% DPPH and 12.9% hydroxyl radical scavenging at 3 mg/mL EPS. These findings highlight *S. thermophilus* GM4 as a safe, technological, and functional candidate for dairy and probiotic applications, with its EPS exhibiting properties suitable for functional food incorporation.

## 1. Introduction

*Streptococcus thermophilus* is a lactic acid bacterium (LAB) of significant industrial importance, particularly within the dairy industry. It is widely employed as a starter culture in the production of fermented milk products, including yogurt and cheese. Its rapid acidifying capacity during milk fermentation is essential for the coagulation of milk proteins, a critical step in dairy processing. Recognized globally for its technological roles, *S. thermophilus* holds Generally Recognized as Safe (GRAS) status, confirming its long-established safety for consumption [1,2].

Beyond its primary function in acidification, certain strains of *S. thermophilus* are capable of producing exopolysaccharides (EPSs)—high-molecular-weight polymers composed of repeating sugar units, synthesized and secreted by the bacterium. EPSs play a crucial role in determining the texture, viscosity, and overall sensory quality of fermented dairy products [3]. The increasing societal demand for natural and functional food ingredients has further elevated the importance of microbial EPSs, particularly those derived from well-characterized and safe organisms such as *S. thermophilus* [1]. This Gram-positive, thermophilic bacterium grows optimally at 35–42 °C and is classified as a facultative anaerobe, primarily producing lactic acid via fermentation [4]. In yogurt production, *S. thermophilus* exhibits a well-documented symbiotic relationship with *Lactobacillus delbrueckii* subsp. *bulgaricus*, in which it provides essential metabolites such as folic and formic acids that support purine synthesis by its partner organism [5].

In addition to acidification, *S. thermophilus* contributes to various desirable qualities in dairy products through the production of metabolites including EPS, aromatic compounds, and vitamins such as folic acid. These metabolites enhance texture, viscosity, flavor, and water-holding capacity, all of which are vital for consumer acceptance [1].

*S. thermophilus* is also considered a probiotic candidate due to its ability to survive gastrointestinal transit and contribute to gut health [6]. It assists in lactose digestion, making dairy products more tolerable for lactose-intolerant individuals, and has been associated with potential health benefits such as anti-inflammatory, antioxidant, antimicrobial, and immunomodulatory activities [6]. It is also suggested that it has a role in lowering serum cholesterol levels [7]. The dual functionality of *S. thermophilus*—as an industrial starter culture and a potential probiotic—underscores the significance of its metabolic products, particularly EPS.

EPS production in *S. thermophilus* is a strain-dependent trait. While some strains produce large quantities of EPS, others synthesize little to none, highlighting the need for targeted screening and the selection of strains for specific technological applications [8]. Two main forms of EPS are produced: capsular EPSs, which remain attached to the cell surface, and free EPSs, which are secreted into the surrounding medium. Free EPSs can further be categorized based on their impact on product texture—ropy EPSs create a stringy, viscous texture, while non-ropy EPSs still contribute to viscosity without the stringiness [9,10].

The composition and concentration of the growth medium, especially the type of carbon source (e.g., glucose, lactose, sucrose), significantly influence both bacterial growth and EPS production [11]. This highlights the importance of optimizing fermentation conditions to enhance EPS yield and tailor its functional properties for specific applications. Additionally, some EPSs possess bioactive properties which can enhance the functional and preservative qualities of food [5,12].

In this study, four *S. thermophilus* strains (GM1 to GM4) were isolated from raw goat milk collected in the Azores, Portugal. Preliminary analysis revealed their ability to produce ropy EPSs [13]. This work aims to characterize these strains, with a focus on their technological properties for dairy fermentation, safety, and probiotic potential. Additionally, the EPS produced by strain GM4 (which lacks virulence factors) was purified and characterized to assess its potential applications in food.

## 2. Materials and Methods

### 2.1. Materials

The following materials were used: MRS broth and agar (Biokar Diagnostics, Allonne, France), skim milk (VWR Chemicals, Leuven, Belgium), PCA (Biokar Diagnostics, France), M17 (Biokar, France), MSE (Biokar, France), Rogosa (Biokar, France), Muller–Hinton agar (AES, Bruz, France), Tributyrin agar (Merck, Darmstadt, Germany), Potato dextrose agar (PDA, HiMedia, Mumbai, India), Tryptose Blood Agar Base (Merck Germany), DNAse-Test agar (Sigma-Aldrich, St. Louis, MO, USA), glucose (Sigma, Saint-Quentin-Fallavier, France), lactose (Scharlau, Barcelona, Spain), fructose (Sigma, Burlington, MA, USA), sucrose (Sigma, Darmstadt, Germany), HCl (PanReac, Darmstadt, Germany), TCA (PanReac AppliChem ITW reagents, Darmstadt, Germany), Peptone (Fluka, Newport News, VA, USA), Yeast extract (AES, Bruz, France), Gelatine (Biolife, Milan, Italy), Bacteriological agar (AES, Bruz, France), APIZYM kit (Cat. No. 25200, Bio-Mérieux, Marcy-l’Étoile, France), DNAse type I (Amresco, Solon, OH, USA), pronase E (Amresco, Solon, OH, USA), DPPH (Sigma, Darmstadt, Germany), brilliant green (Sigma, New Delhi, India), ascorbic acid (Riedel-de Haën, Seelze, Germany), cholesterol (Sigma-Aldrich, USA), methanol (Fisher Chemical, Loughborough, UK), PBS (Sigma, Saint Louis, MO, USA), Fe_2_SO_4_ (Riedel-de Haën, Berlin, Germany), H_2_O_2_ (Panreac Química SAU, Barcelona, Spain), dialysis membrane (10 kDa, Cat. No. 11405929, Spectrum Laboratories Inc., Piscataway, NJ, USA), antimicrobial susceptibility discs (Oxoid, Thermo Scientific, Basingstoke, UK), UltraClean^®^ Microbial DNA Isolation Kit (MoBio, Carlsbad, CA, USA), and Sybergreen (Cat. No. S33102, Invitrogen, Carlsbad, CA, USA).

### 2.2. Bacterial Isolation and Identification

The strains *S. thermophilus* GM1, GM2, GM3, and GM4 were isolated from goat milk (The Azores, Portugal). Briefly, goat milk samples were collected from their disinfected mammary glands, taking care to discard the first three jets. The samples were 10-fold serially diluted with peptone water, spread onto de Man Rogosa Sharpe (MRS) agar, and incubated at 37 °C for 72 h. Pure cultures were obtained through ≥3 subcultures under identical conditions. Single colonies were then aerobically grown in MRS broth (37 °C, 24 h). Cells (1 mL aliquots) were pelleted by centrifugation (14,000× *g*, 2 min), and genomic DNA was extracted from the pellet using the Microbial DNA Isolation Kit (UltraClean^®^) following manufacturer instructions. The isolates (4) were identified via sequencing of the 16S rRNA gene, as described by Pires et al. [14]. PCR-amplified fragments were sequenced (STAB Vida, Caparica, Portugal) and analyzed via NCBI BLAST (http://www.ncbi.nlm.nih.gov/, accessed on 20 September 2022). The 16S rDNA sequences were deposited in the GenBank under accession numbers OQ540791, OQ540792, OQ540793, and OQ540794, respectively. Stock cultures were kept at −80 °C in 30% (*v*/*v*) glycerol and propagated twice in MRS broth with 1% (*v*/*v*) of inoculum at 37 °C for 24 h.

### 2.3. Technological Characterization of S. thermophilus Strains

#### 2.3.1. Production of EPS with Different Sugar Substrates

The bacterial cultures were grown for 48 h at 37 °C in modified MRS broth where glucose was replaced by 10% (*w*/*v*) of either of the following sugars: lactose, sucrose, glucose, or fructose [15]. After the incubation period, the tubes were centrifuged (4000× *g*, 20 min, 4 °C). The EPS was extracted from the supernatant with two volumes of 95% (*v*/*v*) cold ethanol. The precipitation step was repeated twice, before EPS precipitates were dissolved in warm ddH_2_O. The total sugar content of EPS was evaluated by the phenol sulfuric acid method [16], using glucose as standard.

#### 2.3.2. Milk Acidification

The strains were inoculated into MRS broth and incubated at 37 °C for 24 h. After this incubation, a 1% inoculum was prepared in skim milk and incubated again at 37 °C for 24 h. The pH was measured at 0, 6, and 24 h.

#### 2.3.3. Carbohydrate Fermentation

To assess the ability to ferment different carbohydrates, a 1% solution of carbohydrates was tested: glucose, fructose, galactose, ribose, arabinose, sucrose, lactose, maltose, raffinose, starch, mannitol, xylose, trehalose, rhamnose, dextrin, sorbitol, glycerol, and inulin. A suspension of each strain was prepared in 10 mL of API 50 CHL suspension medium (10 g/L polypeptone, 5 g/L yeast extract, 0.1% Tween 80, 2 g/L dipotassium phosphate, 5 g/L sodium acetate, 2 g/L diammonium citrate, 0.2 g/L magnesium sulfate, 0.05 g/L manganese sulfate, 0.17 g/L bromcresol purple) and the optical density adjusted to 2 on the McFarland scale. The bacterial suspension (400 μL) was then added to 100 μL of each sugar, covered with mineral oil and incubated at 37 °C for 48 h. The fermentation was evaluated by the acidification of the medium, which was visible by the color change of the indicator (from blue to yellow).

#### 2.3.4. Enzymatic Activity

The APIZYM kit was used as a semi-quantitative method to detect the enzymatic activities of 19 enzymes: alkaline phosphatase, esterase (C4), esterase–lipase (C8), lipase (C14), leucine arylamidase, valine arylamidase, cystine arylamidase, trypsin, *α*-chymotrypsin, acid phosphatase, naphthol-AS-BI phosphohydrolase, *α*-galactosidase, *β*-galactosidase, *β*-glucuronidase, *α*-glucosidase, *β*-glucosidase, N-acetyl-*β*-glucosaminidase, α-mannosidase, and α-fucosidase [17]. The experimental procedure was made according to the manufacturer’s instructions. Results were expressed as nmol of hydrolyzed substrate, determined from reaction intensity measurements using a quantitative scale ranging from 0 (no activity) to 5 (≥40 nmol hydrolyzed substrate).

#### 2.3.5. Proteolytic Activity

Extracellular proteolytic activity was determined by plating the isolates on Plate Count Agar (PCA) supplemented with 10% (*w*/*v*) skim milk. The isolates were then incubated at 37 °C and after 72 h the plates were flooded with 1% HCl. Proteolytic activity was indicated by a clear zone around the colonies [18]. *Staphylococcus aureus* subsp. *aureus* ATCC 25923 was used as a positive control.

#### 2.3.6. Lipolytic Activity

To evaluate the lipolytic activity, the LAB were inoculated on Tributyrin agar and incubated at 37 °C for 72 h. After incubation, the presence of colonies surrounded by a clear halo indicated a positive result [19]. *Staph. aureus* ATCC 25923 was used as a positive control.

### 2.4. Safety Evaluation of S. thermophilus Strains

#### 2.4.1. Hemolytic Activity

The strains were inoculated onto blood agar medium prepared from Tryptose Blood Agar Base with the addition of sheep’s blood. The plates were incubated at 37 °C for 48 h. After this time, the blood agar plates were analyzed and classified as follows: *β*-hemolysis, when a translucent halo appeared around the colonies; α-hemolysis, corresponding to a greenish halo around the colonies; and *γ*-hemolysis, indicating the absence of a halo around the colonies [20]. *Staph. aureus* ATCC 25923 was used as a positive control.

#### 2.4.2. DNAse Activity

DNAse enzyme production was analyzed using DNAse-Test agar. After incubation for 48 h at 37 °C, the results were recorded. A positive reaction was indicated by the appearance of a pink halo around the colonies [21]. *Staph. aureus* ATCC 25923 was used as a positive control.

#### 2.4.3. Gelatinase Activity

The gelatinase activity was determined according to the methodology of Terzić-Vidojević et al. [22]. A culture medium was prepared containing 5 g/L peptone, 3 g/L yeast extract, 30 g/L gelatine, and 17 g/L agar (pH 7.0) After incubation at 37 °C for 48 h, the inoculated plates were flooded with a saturated ammonium sulphate solution. In this test, the appearance of a transparent halo around the colonies is considered an indicator of gelatin degradation by gelatinase, indicating a positive reaction. *Staph. aureus* ATCC 25923 was used as a positive control.

#### 2.4.4. Antibiotic Susceptibility

Antibiotic resistance was assessed using the Kirby–Bauer method (agar diffusion). First, 100 µL LAB (probably the bacterial lactic acid cultures from the previous steps) was inoculated into 5 mL MRS broth and incubated at 37 °C for 24–48 h. After this incubation time, the optical density (OD) was adjusted to a 0.5 McFarland standard. Subsequently, 400 µL of this suspension was transferred to a new tube containing 5 mL of MRS broth. The contents of each tube were then evenly distributed onto two plates of Muller–Hinton agar. The plates were allowed to stand for approximately 25 min to allow the agar to absorb some of the liquid. Excess liquid was discarded, and the plates were allowed to dry thoroughly in a laminar flow cabinet for 1 h. The antibiotic discs were then applied to the agar surface using sterile forceps. Ten antibiotics were tested: ampicillin (10 µg), oxacillin (1 µg), vancomycin (30 µg), gentamicin (10 µg), tetracycline (30 µg), chloramphenicol (30 µg), streptomycin (10 µg), erythromycin (15 µg), clindamycin (2 µg), and kanamycin (30 µg). The discs were positioned so that the inhibition zones did not overlap, six discs per plate. The discs were incubated at 37 °C for 24–48 h. After incubation, the zones of inhibition, including the disc diameter, were measured using a ruler. *Staph. aureus* ATCC 25923 was used as positive control. The reference values from CLSI [23] were used to interpret the results.

#### 2.4.5. Virulence Genes

PCR was used to detect genes related to virulence (*gelE*, *hyl*, *asa1*, *esp*, *cylA*, *efaA*, *ace*), antibiotic resistance (*vanA*, *vanB*), and biogenic amine production (*hdc1*, *hdc2*, *tdc*, *odc*) as described by Ribeiro et al. [24]. Genomic DNA was extracted from pure cultures as described in Section 2.2. PCR was performed using a Thermocycler TProfessional Basic 96 (Biometra, Göttingen, Germany) with primers and expected fragment sizes detailed in Supplementary Table S1. Amplified products were resolved on a 2% agarose gel in 0.5 × TAE buffer and stained with SybrGreen (15 µg/mL). Positive controls included genomic DNA from reference *E. faecalis* and *E. faecium* strains (IITAA collection) [14].

### 2.5. Potential Probiotic Properties of S. thermophilus GM4

#### 2.5.1. Gastrointestinal Resistance

Two tests were performed to evaluate resistance to gastrointestinal conditions: resistance to pH 2.5 (to evaluate acidic gastric conditions) and resistance to bile acids and pancreatin (resistance to intestinal conditions) [14]. The isolates were inoculated in MRS broth and incubated at 37 °C for 24 h. After this time, the culture media were centrifuged in Falcon tubes at 4000× *g* for 20 min at 20 °C (Centrifuge 5804R, Eppendorf, Germany). The pellet was washed with PBS buffer (pH 7.2) and centrifuged under the same conditions. The bacterial pellet was then diluted in 1 mL PBS to obtain the bacterial suspension. One hundred μL of this suspension was placed in two tubes: one tube contained 9.9 mL of a PBS solution with a pH of 2.5 (gastric conditions) and the other tube contained 9.9 mL of a PBS solution (pH 7.2) with 0.3% (*w*/*v*) bile salts and 0.1% pancreatin. The tubes were incubated at 37 °C for 1, 2, and 3 h, reflecting the time the food spends in the stomach and intestine. After the indicated times, viable bacteria were counted by decimal dilution in peptone water and plating on MRS agar.

#### 2.5.2. Mucin Adhesion

The mucin-binding ability of *S. thermophilus* GM4 was evaluated using a 96-well plate method as described by Jurášková et al. [25]. Porcine mucin (0.5 mg/mL in PBS) was used to coat wells overnight at 4 °C, followed by PBS washes to remove unbound mucin. Bacterial cells (10^8^ CFU/mL in PBS) were added to the wells and incubated at 37 °C for 90 min. Non-adherent cells were removed by repeated PBS washes, and adherent cells were released using 0.05% Triton X-100. Viable counts were determined by plating on MRS agar and incubating at 37 °C for 48 h. The probiotic *Lacticaseibacillus rhamnosus* GG (ATCC 53103) was also tested for comparison.

#### 2.5.3. Auto-Aggregation

Auto-aggregation was measured according to the method described by Todorov and Dicks [26]. The strain was inoculated in MRS broth and incubated at 37 °C for 24 h. After this time, the cultures were centrifuged at 4000× *g* for 20 min at 20 °C. The bacterial pellet was washed with PBS and centrifuged again under the same conditions. PBS was added to the pellet and the initial absorbance (Abs_initial_) was measured at 600 nm. After 60 min, the cultures were centrifuged again at 300× *g* for 2 min at 20 °C and the final absorbance (Abs_final_) was measured. This test was performed in quadruplicate. Auto-aggregation was determined using the following equation:

$$Auto\text{-}agregation\;\left(\%\right)=\;\frac{\left({Abs}_{initial}-{Abs}_{final}\right)}{{Abs}_{initial}}\times \;100$$

#### 2.5.4. Co-Aggregation

The ability of *S. thermophilus* to co-aggregate with pathogenic bacteria was analyzed according to the methodology described by Chen et al. [27]. The following pathogenic bacteria were used for this test: *Escherichia coli* ATCC 25922, *Listeria monocytogenes* ATCC 13932, *Staph. aureus* ATCC 25923, and *Salmonella enterica* subsp. *enterica* serovar Typhimurium ATCC 14028. Co-aggregation was determined using the following equation:

$$Co\text{-}aggregation\;\left(\%\right)=\;\frac{\left[\left(A_{x}+A_{y}\right)/\left(2-A_{x+y}\right)\right]}{A_{x}+A_{y}}\;\times \;100$$

A_x_ and A_y_ represent the absorbance (600 nm) of the two bacteria cell suspensions and A_x+y_ means the absorbance of mixed bacteria cell suspensions.

### 2.6. Functional Properties of S. thermophilus GM4

#### 2.6.1. *β*-Galactosidase Activity

The β-galactosidase activity of strain GM4 was assessed based on Miller’s method, with slight adjustments [28]. The strain was cultured overnight in MRS broth at 37 °C, and its optical density was measured at 600 nm. For the assay, 20 µL of bacterial culture was mixed with 80 µL of freshly prepared permeabilization solution (containing 100 mM Na_2_HPO_4_, 20 mM KCl, 2 mM MgSO_4_, 0.8 mg/mL CTAB, 0.4 mg/mL sodium deoxycholate, and 5.4 μL/mL β-mercaptoethanol added just before use). Then, 600 µL of substrate solution (1 mg/mL ONPG in phosphate buffer, pH 7.0, with 2.7 μL/mL *β*-mercaptoethanol added freshly) was added. The mixture was incubated at 37 °C for around 30 min or until a yellow color developed. The reaction was stopped with 700 µL of 1 M Na_2_CO_3_ and centrifuged at 16,100× *g* for 5 min (Eppendorf model 5415D, Eppendorf, Hamburg, Germany). The absorbance of the supernatant was read at 420 nm to calculate *β*-galactosidase activity.

$$\beta \text{-}galactosidase\;activity\left(Miller\;units\right)=1000\times \frac{Abs420}{\left(Abs600\times 0.2\times reaction\;time\right)}$$

#### 2.6.2. Cholesterol-Lowering Ability

The cholesterol-reducing ability of GM4 was evaluated in vitro using MRS broth supplemented with 0.5 mg/mL cholesterol [29]. The medium was inoculated with 1% log-phase bacterial culture and incubated at 37 °C for 24 h. The remaining cholesterol in the medium was measured enzymatically (CHOD-PAP method, NS Biotec, Alexandria, Egypt), and the reduction percentage was calculated as:

$$Cholestero\;reducing\;ability\left(\%\right)=\;100\;\times \;\frac{{Cholesterol}_{initial}-\;{Cholesterol}_{final}}{{Cholesterol}_{initial}}$$

#### 2.6.3. DPPH Scavenging Activity

The antioxidant activity was determined using the DPPH method according to Aarti and Khusro [30], with some modifications. Overnight cultures were centrifuged and resuspended in dipotassium hydrogen phosphate buffer. The cells were added to 1 mL of a 0.2 mM DPPH solution and incubated for 30 min in the dark at room temperature. A methanol-treated sample served as a blank, and DPPH without bacteria was the control. Ascorbic acid was used as positive control. The absorbance was measured at 517 nm to determine the scavenging ability.

$$DPPH\;scavenging\;activity\;\left(\%\right)=\left[1-\left(\frac{A_{sample}-A_{blank}}{A_{control}}\right)\right]\times 100$$

#### 2.6.4. Hydroxyl Scavenging Activity

To evaluate the degradation of hydroxyl radicals, the method of He et al. [31] was adapted. The reaction mixture contained 1 mL 0.5 mM Brilliant Green, 2 mL 0.5 mM FeSO_4_, and 1.5 mL H_2_O_2_. The bacterial cells were centrifuged and added to the mixture, which was incubated for 15 min at room temperature. The absorbance was then measured at 624 nm. Ascorbic acid was used as positive control.

$$Hydroxyl\;radical\;scavenging\;activity\;\left(\%\right)=\left[\frac{A_{1}-A_{0}}{A-A_{0}}\right]\times 100$$

#### 2.6.5. Production of EPS in Skim Milk

EPS production was investigated in skim milk supplemented with 10% lactose. A 1% bacterial inoculum was grown in MRS broth overnight at 37 °C and then centrifuged at 3500× *g* for 30 min at 4 °C. The pellet was resuspended in skim milk with 10% lactose or without addition of this sugar (control), and incubated at 37 °C for 48 h. During fermentation, the pH, titratable acidity, and viable counts in MRS (CFU/mL) were measured (at 0 h, 2 h, 4 h, 6 h, 24 h, 48 h). To extract EPS, 25 mL of the fermented medium was diluted 1:1 with MilliQ water. The casein was precipitated with 4 mL of 0.2 M TCA and removed by centrifugation (3500× *g*, 30 min, 4 °C). The supernatant was adjusted to a pH of 6.8 with 4 M NaOH, then boiled for 30 min and centrifuged again to remove the whey proteins. An equal volume of cold ethanol was added to the supernatant to precipitate EPS at 4 °C overnight. The resulting pellet was resuspended in 10 mL of MilliQ water, sonicated for 1 h, and vortexed until uniform. The solution was dialyzed against MilliQ water for 7 days at 4 °C (the water was changed twice daily) and then stored at −20 °C for further analysis. The EPS concentration was determined using the phenol sulfuric acid method [16].

### 2.7. Purification and Characterization of EPS from S. thermophilus GM4

#### 2.7.1. Purification of EPS

EPS was produced using a method described by Domingos-Lopes et al. [32], with modifications. *S. thermophilus* GM4 (1% inoculum in 50 mL MRS broth) was grown overnight at 37 °C, then transferred to 1 L of modified MRS broth supplemented with 10% (*w*/*v*) of test sugars (glucose, fructose, sucrose, or lactose) for 48 h fermentation at 37 °C. Cells were removed by centrifugation (9000× *g*, 5 min, 4 °C), and EPS was precipitated from the supernatant using two volumes of cold ethanol (4 °C, 48 h). After recovery centrifugation (10,000× *g*, 20 min, 4 °C), the pellet was dissolved in MilliQ water, dialyzed (3 days, 4 °C; water changed twice daily), and freeze-dried. The entire process was repeated four times to ensure sufficient material for EPS yield quantification, which was performed using the phenol sulfuric acid method.

For further purification to analyze the EPS composition and linkage, the crude EPS was dissolved at a concentration of 5 mg/mL in a buffer solution (50 mM Tris–HCl, 10 mM MgSO_4_.7H_2_O, pH 7.5) and treated with DNAse type I (2.5 μg/mL) for 6 h at 37 °C to remove any DNA. Subsequently, Pronase E (50 μg/mL in 50 mM Tris–HCl, 2% EDTA, pH 7.5) was added, and the mixture was incubated for 18 h at 37 °C to degrade any proteins present. This purified EPS was used for detailed compositional and linkage analysis.

#### 2.7.2. EPS Purity

EPS moisture and ash content was determined using thermogravimetric analysis (Setaram Setsys Evolution 1750 TGA, Setaram, Lion, France) under a nitrogen atmosphere (200 mL/min) [33]. Total sugar content was quantified by the phenol sulphuric acid method [16] and protein was assayed by the method of Bradford [34].

#### 2.7.3. Carbohydrate Composition

Samples were analyzed for their neutral sugar content after acid hydrolysis, derivatization to alditol acetates, and analysis of individual neutral sugars and glycosamines by gas chromatography with flame ionization detector (GC-FID) and coupled to mass spectrometry detector (GC-MS). To identify uronic acids, hydrolyzed exopolysaccharides were analyzed using high-performance anion exchange chromatography with pulsed amperometric detection (HPAEC-PAD) [33].

The total sugars were determined by the sum of the amount of the individual sugars, considering that the hydrolysis of a glycosidic linkage results in an addition of a water molecule into the sugar structure.

##### Neutral Sugar Analysis by GC-FID

EPS (1 mg) was hydrolyzed in 2 M TFA (120 °C, 1 h). After cooling and TFA evaporation (speedvac), the hydrolysate was reconstituted in water (1 mg/mL). A 60 μL aliquot was mixed with 80 μL internal standard (2-deoxyglucose, 0.1 g/L) and 100 μL of 25% NH_3_. Samples were reduced with NaBH_4_ (15% in 3 M NH_3_; 1 h, 30 °C) and acetylated with acetic anhydride/1-methylimidazole (30 min, 30 °C) after acidification. Alditol acetates were extracted into dichloromethane, washed repeatedly with water, and dried under vacuum with anhydrous acetone.

A separate hydrolysis using 1 M H_2_SO_4_ was also tested. Briefly, 1–2 mg of each EPS was weighed into 10 mL tubes, and 200 μL of 72% H_2_SO_4_ was added. After 3 h incubation at room temperature with occasional stirring, 2.2 mL of distilled water was added to achieve a 1 M concentration, and the mixture was incubated for 1 h at 100 °C. The tubes were cooled, and 0.5 mL was removed for subsequent uronic acid analysis. Hydrolysis continued for another 1.5 h at 100 °C, and the neutral sugars were analyzed by GC-FID after reduction and acetylation, as described above.

Alditol acetates from both methods were dissolved in 25 μL of anhydrous acetone and analyzed by GC-FID (Perkin Elmer–Clarus 400) equipped with a 30 m DB-225 column (i.d. 0.25 mm, film thickness 0.15 μm, J&W Scientific, Folsom, CA, USA). The oven temperature was held at 220 °C for 7 min, then increased to 240 °C at 20 °C/min and held for 1 min. The injector and detector temperatures were 220 °C and 240 °C, respectively, with an H_2_ carrier gas flow rate of 1.7 mL/min.

Alditol acetates were also analyzed by GCqMS (GC-2010 Plus, Shimadzu, Japan) using a non-polar ZB-5HT INFERNO column (30 m × 0.25 mm × 0.10 μm). A 0.5 μL sample was injected in split mode at 250 °C. The oven temperature program was: 140 °C to 180 °C at 5 °C/min, hold 1 min; 180 °C to 250 °C at 5 °C/min; and 250 °C to 350 °C at 100 °C/min. The He carrier gas flow rate was 0.86 mL/min. The mass spectrometer operated in electron impact (EI) mode at 70 eV, scanning *m/z* 50–400 in full-scan mode.

##### Uronic Acid Analysis

Uronic acids were determined by the *m*-phenylphenol colorimetric method, using galacturonic acid (GalA, 10 to 100 μg/mL) as standard. To 100 μL of the diluted sample hydrolyzed with 1 M H_2_SO_4_ (1:4) were added 1 mL of 200 mM boric acid in 96% (*w*/*w*) H_2_SO_4_. After vortexing for 5–7 s, the test tubes were heated at 100 °C, across 10 min. After cooling, 20 μL of *m*-phenylphenol was added, the test tubes were vortexed, and 300 μL was transferred to well plates in duplicate, performing with a reaction time of 15–30 min before the absorbance was read at 520 nm.

##### Carbohydrate Analysis by HPAEC-PAD

HPAEC-PAD analysis was performed as described by Circuncisão et al. [35] on a Dionex ICS-6000 system equipped with an AS-AP autosampler, an SP pump, a DC chromatography oven, and an electrochemical detector fitted with a permanent gold working electrode and an AgCl reference electrode. The system was controlled by Chromeleon 7.3 software (Thermoscientific Dionex, Sunnyvale, CA, USA), and detection was achieved using integrated amperometry with the standard quadruple waveform for carbohydrates. Separation was achieved using a Dionex CarboPac PA100 analytical column (250 × 4 mm) protected by a corresponding guard column (50 × 4 mm), maintained at 30 °C, with a constant flow rate of 1.0 mL/min. The eluents consisted of MilliQ water (eluent A), 500 mM NaOH (eluent B), and 500 mM sodium acetate (eluent C). Prior to analysis, EPS samples (1–2 mg) were hydrolyzed in 2 M TFA for 1 h at 120 °C in sealed tubes.

The hydrolysate was evaporated to dryness, reconstituted in MilliQ water to a final concentration of 1 mg/mL, and filtered through a 0.2 µm nylon membrane. A 25 µL aliquot of the prepared sample was injected and eluted using a multi-step gradient: initial conditions were held at 100% A for 40 min, followed by a linear gradient to 82:3:15 (A:B:C) over 5 min, which was held isocratically for a further 10 min. This was followed by a 5 min step to 0:75:25, a 10 min wash with 100% B, and finally, a 15 min re-equilibration to initial conditions. Standards of neutral sugars, aldobiouronic acid from arabic gum, and uronic acids were used to confirm or characterize the carbohydrate composition of EPS.

#### 2.7.4. Methylation Analysis

Glycosidic linkage composition of EPS was analyzed by methylation [36]. EPS (1–2 mg) was dissolved in 1 mL of anhydrous dimethyl sulfoxide and then NaOH (40 mg) was added under argon. The samples were methylated by the addition of 80 µL of CH_3_I and stirring (20 min). CHCl_3_/MeOH (1:1, *v*/*v*, 3 mL) was added, and the solution was dialyzed using membranes with a 12 kDa cut-off against 3 lots of 1 L of 50% EtOH. The dialysate was evaporated to dryness and the material was remethylated using the same procedure. The methylated polysaccharide was hydrolyzed with 2 M TFA for 1 h at 120 °C. After evaporation of TFA, the hydrolyzed methylated sugars were reduced with sodium borodeuteride (NaBD_4_) and acetylated as described for the neutral sugar analysis by GC-FID (Section Neutral Sugar Analysis by GC-FID).

The partially methylated alditol acetates were separated and analyzed by gas chromatography quadrupole mass spectrometry (GCqMS, GC-2010 Plus, Shimadzu, Japan) using a non-polar column HT5 (50 m × 0.22 mm × 0.10 µm, Trajan, Australia), with He carrier gas (1.75 mL/min) and the injection of 0.5 μL in split mode (at 250 °C). The GC oven temperature program was set to start at 110 °C, raised to 170 °C at 7.5 °C/min, holding for 4.93 min, raised to 174 °C at 0.20 °C/min, raised to 250 °C at 15 °C/min, and holding for 5 min. The mass spectrometer was operated in the electron impact mode (EI) at 70 eV scanning the range 70–370 *m*/*z*, in a full-scan acquisition mode. Chromatogram peaks were identified comparing all mass spectra with a laboratory-made database and the Complex Carbohydrate Research Center Spectral database (https://glygen.ccrc.uga.edu/ccrc/specdb/ms/pmaa/pframe.html, accessed on 6 August 2025).

#### 2.7.5. Molecular Weight Distribution and Homogeneity

The molecular weight (Mw), number-average weight (Mn), and polydispersity index (Mw/Mn) of EPS were estimated using two PL aquagel-OH MIXED 8 µm 300 × 7.5 mm columns protected by a PL aquagel-OH Guard 8 µm 50 × 7.5 mm pre-column on a Malvern Panalytical system (UK) on OMNISEC v11.41 software. The columns and the refractive index detector were maintained at 40 °C during the analysis and the autosampler was maintained at 35 °C. The eluents (water and 0.1 M NaNO_3_) were pumped at a flow rate of 0.9 mL/min. The columns were calibrated with pullulans (Polymer Laboratories, Shropshire, UK) in the range 5.8–380 kDa (calibration curve: logM = 74.262 − 11.199x + 0.626x^2^ − 0.012166x^3^, R^2^ = 0.998). EPS was dissolved in a 0.1 M NaNO_3_ aqueous solution at 25 °C for 60 min to reach a 2–4 mg/mL concentration and filtered through a 0.22 µm PTFE filter [37].

#### 2.7.6. Dextranase Resistance

The EPS purified from GM4 was tested for dextranase resistance using 1,6-*α*-D-glucan 6-glucanohydrolase from *Chaetomium erraticum* (D0443, Sigma), as reported by Domingos-Lopes et al. [32].

#### 2.7.7. DPPH Scavenging Activity

The DPPH radical scavenging ability of EPS was evaluated following the method of Hu et al. [38]. The EPS was dissolved in distilled water at concentrations of 1, 2, and 3 mg/mL. For the assay, 2 mL of 0.1 mM DPPH solution was mixed with 2 mL of the EPS solution (denoted as A_1_) and incubated in the dark for 30 min. To account for background absorbance, the DPPH solution was replaced with ethanol, and 2 mL of the EPS solution was added to measure A_2_. As a control, 2 mL of distilled water was mixed with 2 mL of DPPH solution (denoted as A_0_). Ascorbic acid (1 mg/mL) was used as a positive control. The DPPH scavenging activity was calculated using the following formula:

$$DPPH\;scavenging\;activity\;\left(\%\right)=\;\left[1-\left(\frac{A_{1}-\;A_{2}}{A_{0}}\right)\right]\;\times \;100$$

#### 2.7.8. Hydroxyl Scavenging Activity

The hydroxyl radical scavenging capacity of EPS was determined using a modified version of the Fenton reaction [39]. The reaction mixture included 100 µL of 0.435 mM Brilliant Green, 200 µL of 0.5 mM FeSO_4_, 150 µL of 3.0% H_2_O_2_, and 100 µL of EPS solution. The mixture was incubated at room temperature for 20 min, then centrifuged at 5000× *g* for 5 min before measuring absorbance at 624 nm. The scavenging activity was calculated using the following equation:

$$Hydroxyl\;radical\;scavenging\;activity\;\left(\%\right)=\;\left[\frac{A_{s}-\;A_{0}}{A-A_{0}}\right]\;\times \;100$$

As with the absorbance of the sample-containing mixture, A_0_ is the absorbance of the mixture with distilled water instead of the sample, and A is the absorbance of the complete Fenton reaction system without any sample. Ascorbic acid (1 mg/mL) was used as a positive control.

### 2.8. Statistical Analysis

All experiments were conducted in triplicate, with results presented as mean ± standard error of the mean (SEM). Data were analyzed using one-way ANOVA followed by Tukey’s post hoc test to identify significant differences (*p* < 0.05) between treatment groups. All statistical analyses were performed using SPSS software (version 30; SPSS Inc., Chicago, IL, USA).

## 3. Results and Discussion

### 3.1. Technological Characterization of S. thermophilus Strains

#### 3.1.1. Production of EPS with Different Sugar Sources

**Figure 1:**
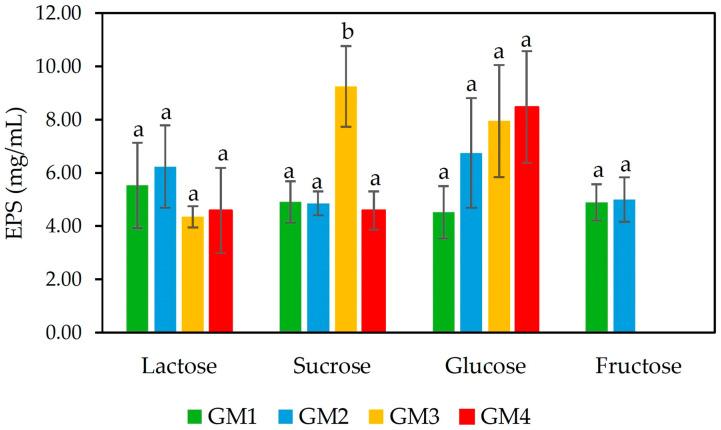
EPS production by *S. thermophilus* GM1, GM2, GM3, and GM4 in mMRS supplemented by different carbohydrate sources: lactose, sucrose, glucose, and fructose. Within each sugar, different letters indicate a significant difference between strains (*p* < 0.05).

Figure 1 presents EPS production by *S. thermophilus* strains isolated from goat milk using different sugar sources. While the strains generally exhibited high EPS yields in glucose, sucrose, and lactose, their ability to utilize fructose varied. Two strains (GM1 and GM2) produced EPS across all tested substrates, including fructose. However, the failure of strains GM3 and GM4 to produce EPS in fructose indicates that sugar source may play a role in EPS production for some strains. These divergent results imply that while sugar type is not restrictive for the ropy phenotype, strain-specific metabolic differences likely influence substrate preferences.

The highest EPS yields (4.6–9.2 mg/mL) were observed in 10% sucrose, followed by glucose (4.5–8.5 mg/mL), lactose (4.3–6.2 mg/mL), and fructose (4.9–5.0 mg/mL). Strain GM2 achieved its maximum EPS production (9.2 mg/mL) in sucrose-supplemented MRS medium, highlighting sucrose as the most effective carbon source for this strain. In contrast, GM4 produced higher EPS levels in glucose, with comparable yields in lactose and sucrose.

These findings are consistent with prior studies indicating that sucrose is the preferred carbon source for EPS production by *S. thermophilus*, followed by glucose and lactose [4]. The strains under study produced a higher EPS content than *S. thermophilus* ASCC 1275 (0.430 mg/mL in M17 medium supplemented with 1% sucrose) [40], and *S. thermophilus* ST1 (0.1358 mg/mL in skim milk containing 2% sucrose) [10]. However, some strains exhibit divergent preferences, with lactose leading to higher yields than sucrose [41,42], as with *S. thermophilus* LY03, which produced higher EPS with lactose than glucose [43], and *S. thermophilus* JM905, which favored lactose as a carbon source [44].

Genomic and biochemical analyses confirm that *S. thermophilus* can utilize various sugars for EPS synthesis, though molecular mass and yield vary depending on the carbon source [45]. The optimal substrate for EPS production is strain-dependent, with sucrose and lactose often outperforming other sugars, though exceptions exist [41,42].

#### 3.1.2. Carbohydrate Fermentation

**Figure 2:**
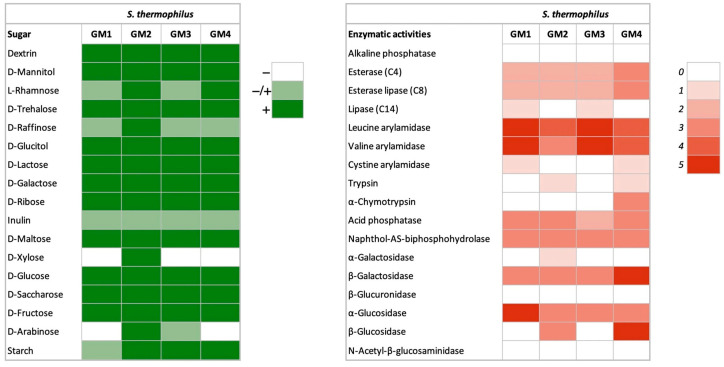
Heatmap of *S. thermophilus* GM1, GM2, GM3, and GM4 showing (**a**) carbohydrate fermentation (results were classified as negative −, slight positive −/+, and positive +); (**b**) enzymatic activities: ranging from 0 (no activity) to 5 (40 nmol hydrolyzed substrate).

A broad fermentation profile was observed in all four strains of *S. thermophilus* (Figure 2). They showed the ability to ferment lactose, galactose, ribose, maltose, glucose, sucrose, fructose, raffinose, sorbitol (glucitol), mannitol, dextrin, inulin, and starch. This broad substrate utilization indicates their metabolic versatility in a dairy context. The ability of *S. thermophilus* strains to utilize carbohydrates is of great importance in the fermentation of dairy products, as it has a direct effect on the rate of acidification of the milk. Various studies indicate that the utilization of glucose, lactose, and fructose by *S. thermophilus* is consistently observed, while the utilization of sucrose, galactose, and maltose has different profiles [2,46]. Gal-positive strains are of technological importance primarily because of their ability to ferment lactose completely. This results in a lower amount of galactose being present, which cannot serve as a carbon source for spoilage or pathogenic bacteria [47,48].

#### 3.1.3. Enzymatic Activities

The *S. thermophilus* GM1, GM2, GM3, and GM4 exhibited high levels of desirable enzymatic activities, particularly aminopeptidases (leucine and valine arylamidases), *β*-galactosidase, and acid phosphatase (Figure 2). The elevated aminopeptidase activity is a well-documented feature of high-quality *S. thermophilus* starter cultures [49]. These enzymes are of crucial importance for the hydrolysis of peptides from casein into free amino acids. They support bacterial growth in milk and play a crucial role in the development of flavor precursors during fermentation [50]. Such enzymatic activity not only accelerates acidification, which is crucial in the production of yoghurt and cheese, but also improves the sensory quality of the end product [49]. Acid phosphatase is also associated with improved acidification and survivability during cold storage, properties that are beneficial in the commercial processing of dairy products [51]. In addition, galactosidase activity is a probiotic characteristic (high in GM4), as evidence suggests that sufficient bacterial lactase production in the intestinal tract can prevent lactose intolerance symptoms in intolerant individuals [52].

All tested strains lacked detectable N-acetyl-β-glucosaminidase and β-glucuronidase activity—a crucial safety advantage. These enzymes pose significant health concerns as β-glucuronidase can release carcinogenic compounds (e.g., polycyclic aromatic hydrocarbons) through hydrolysis of glucuronides [53], while N-acetyl-β-glucosaminidase reduces N-nitro compounds to mutagenic/carcinogenic amines. Their absence minimizes the potential risks of intestinal toxicity and carcinogenesis [54,55], making these strains particularly suitable for food applications.

#### 3.1.4. Acidification, and Proteolytic and Lipolytic Activities

As shown in Table 1, most *S. thermophilus* strains exhibited a limited pH drop after 6 h of incubation. However, *S. thermophilus* GM1 demonstrated significantly greater acidification (*p* < 0.05) compared to other isolates. By 24 h, strains GM1, GM3, and GM4 showed the most pronounced acidification (ΔpH > 1.5), qualifying them as medium acidifiers. This variability in acidification capacity may be attributed to genetic differences among strains. Previous studies have associated rapid acidification phenotypes with the presence of *prtS*, which encodes a cell envelope proteinase crucial for efficient milk acidification [56].

Among the tested strains, only *S. thermophilus* GM3 and GM4 exhibited proteolytic activity, while none showed lipolytic capacity (Table 1). These findings align with previous reports describing typically weak proteolytic activity in *S. thermophilus*, albeit with strain-dependent variability [45]. Such differences in casein degradation may be attributed to the variable presence of the *prtS* gene, despite the conservation of cytoplasmic peptidases across strains [57]. This limited proteolytic activity explains the common industrial practice of co-culturing *S. thermophilus* with *Lactobacillus delbrueckii* subsp. *bulgaricus* to enhance proteolysis through microbial synergy [57]. Proteolytic activity is an essential trait for *S. thermophilus*, enabling growth in milk by releasing amino acids that fulfill the strict nutritional requirements of this auxotrophic bacterium [46], particularly in pure culture [6]. This activity is also the primary driver of desirable flavors, textures, and bioactive peptides in fermented dairy products [4,49]. However, if uncontrolled, this same activity can lead to significant product defects, including the development of bitter off-flavors and a shortened shelf-life due to persistent protein breakdown [19].

The absence of detectable lipolytic activity in all strains presents a technological advantage for dairy applications, as it eliminates risks of fat degradation and associated off-flavor development [19].

### 3.2. Safety Evaluation of S. thermophilus Strains

#### 3.2.1. Hemolysis, DNAse, and Gelatinase Activity

All four isolates displayed γ-hemolysis (non-hemolytic activity) and tested negative for both DNase and gelatinase production (Table 2). These results confirm a favorable safety profile, supporting the classification of *S. thermophilus* as a non-pathogenic microorganism under the Qualified Presumption of Safety (QPS) by the European Food Safety Authority (EFSA) [58].

#### 3.2.2. Antibiotic Susceptibility

Antibiotic susceptibility profiling (Table 2) was performed according to EFSA guidelines for the assessment of bacteria for resistance to antibiotics of human or veterinary importance [59]. The antibiotics tested included ampicillin, vancomycin, gentamicin, kanamycin, streptomycin, erythromycin, clindamycin, tetracycline, and chloramphenicol, selected for their relevance as critically or highly important antimicrobials in human and animal medicine [59]. Oxacillin was also tested as this β-lactam antibiotic is widely used to treat infections caused by *Staph. aureus* [23].

Distinct resistance profiles were observed among the *S. thermophilus* strains. Contrary to previous reports of common resistance to ampicillin, erythromycin, and tetracycline in dairy isolates [60], all the strains tested were susceptible to ampicillin, and resistance to the latter two antibiotics was limited (one strain each). The most frequent resistance was to oxacillin (3/4 strains), with GM2 and GM3 showing the broadest resistance (four antibiotics each). In contrast, the GM4 strain was susceptible to all antibiotics tested. This generally low resistance prevalence aligns with the understanding that significant acquired resistance is uncommon in *S. thermophilus* beyond intrinsic aminoglycoside resistance [61].

Probiotics should not add to the pool of antimicrobial resistance (AMR) genes already present in the gut bacterial population or otherwise increase the spread of AMR.

Although *S. thermophilus* holds GRAS (FDA) and QPS (EFSA) status for food applications, the presence of antibiotic resistance warrants evaluation of potential gene transfer to other bacteria, including pathogens. In this context, strain GM4 emerges as particularly promising for food and probiotic applications due to its complete antibiotic sensitivity profile. However, whole genome sequence analysis is essential to conclusively confirm the absence of transmissible antibiotic resistance genes.

#### 3.2.3. Virulence Genes

The absence of all seven tested virulence genes (*gelE*, *hyl*, *asa1*, *esp*, *cylA*, *efaA*, *ace*, *vanA*, *vanB*, *hdc1*, *hdc2*, *tdc*, and *odc*, Table 2) in the present study is noteworthy, especially when compared to the high prevalence of these determinants reported in several studies [62,63]. Even among foodborne LAB strains, a partial presence of virulence genes has been reported [64,65].

Based on its favorable safety profile—including the absence of virulence factors and antibiotic resistance—*S. thermophilus* GM4 was selected for subsequent probiotic evaluation and EPS characterization. However, its safe application in food may require further validation through animal studies.

### 3.3. Probiotic Evaluation of S. thermophilus GM4

#### 3.3.1. Gastrointestinal Resistance and Adhesion to Mucin

**Figure 3:**
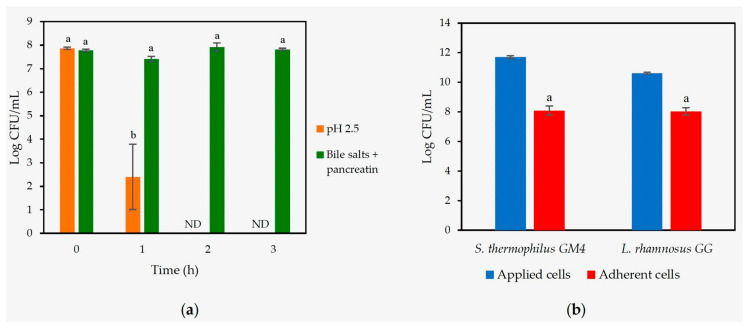
(**a**) Resistance of *S. thermophilus* GM4 to low pH (2.5), bile salts (0.3%, *w*/*v*), and pancreatin (0.1%, *w*/*v*), after 0, 1, 2, and 3 h. ND: not detected. Error bars represent SEM from two experiments; (**b**) adhesion of *S. thermophilus* GM4 and *L. rhamnosus* GG to mucin. Error bars represent SEM from three experiments. Different letters indicate a significant difference (*p* < 0.05).

As shown in Figure 3a, *S. thermophilus* GM4 exhibited moderate but consistent survival under simulated gastrointestinal conditions, with detectable viability after exposure to acidic stress (pH 2.5, 1 h) and intestinal challenges (0.3% bile salts + 0.1% pancreatin, 3 h). These survival levels are comparable to those reported for other *S. thermophilus* strains [66].

*S. thermophilus* GM4 exhibited strong mucin adhesion capability (69.1% binding efficiency), approaching the performance of the probiotic *L. rhamnosus* GG (75.5%) (Figure 3b). This high adhesion capacity, combined with its gastrointestinal stress tolerance, suggests that strain GM4 possesses relevant traits for probiotic applications, though further characterization of its functional properties would be valuable.

#### 3.3.2. Auto-Aggregation and Co-Aggregation

*S. thermophilus* GM4 exhibited strong aggregation properties that enhance its probiotic potential (Table 3). The high auto-aggregation ability (46.8%) is an indicator of cell surface hydrophobicity, a nonspecific interaction that can contribute to bacterial adhesion to cells. This is an important mechanism that promotes intestinal colonization by favoring biofilm formation and adherence to the mucosa, thus improving persistence in the gastrointestinal tract [26]. This property is crucial also for the exclusion of competitors, as probiotic bacteria can physically occupy niches that would otherwise be available to pathogens [67].

Moreover, GM4 exhibited substantial co-aggregation with the key foodborne pathogens *E. coli*, *L. monocytogenes*, and *Staph. aureus* (>30%), along with moderate binding to *S. enterica* (26.4%). This suggests a direct, multi-targeted antagonistic mechanism. Such co-aggregation likely facilitates the physical “capture” and subsequent clearance of pathogens from the gut lumen via peristalsis, effectively reducing their viable population and opportunity to adhere and invade [27].

The observed binding profiles are consistent with established criteria for beneficial probiotic microorganisms, suggesting that *S. thermophilus* GM4 may effectively contribute to the microbial barrier function in the intestinal ecosystem by (1) enhancing its own ecological ability to colonize and (2) acting as a biological scavenger to neutralize a spectrum of pathogens.

### 3.4. Functional Properties of S. thermophilus GM4

#### 3.4.1. β-Galactosidase Activity

The *β*-galactosidase activity of *S. thermophilus* GM4 was remarkably high at 56.2 ± 2.5 × 10^3^ Miller units (Table 4). The *β*-galactosidase activity of GM4 surpassed values reported for *S. thermophilus* ST20 and ST28 [4], and MCC0200 [5]. High enzymatic activity is also documented in *Lactobacillus acidophilus* strains [30]. This is a crucial finding, as *β*-galactosidase is essential for lactose hydrolysis, making it highly relevant for individuals with lactose intolerance. *S. thermophilus* sp. are widely recognized for their contribution to lactose digestion in fermented dairy products like yogurt. For instance, the *β*-galactosidase produced by *S. thermophilus* was shown to remain active during transit through the digestive tract, aiding in lactose breakdown [68,69].

#### 3.4.2. Cholesterol-Lowering Ability

*S. thermophilus* GM4 demonstrated a notable cholesterol-lowering ability (Table 4). This finding aligns with a growing body of research suggesting that certain LAB strains, including *S. thermophilus* strains, can contribute to cholesterol reduction. Several mechanisms are proposed for this effect, including the assimilation of cholesterol by bacterial cells [70]. While some studies on *S. thermophilus* strains report cholesterol removal rates ranging from 2.2% to 6.3% after short incubation periods [71], others, particularly for *Lactobacillus casei* strains, have shown higher reductions, sometimes exceeding 50–60% in simulated gastrointestinal conditions [70].

#### 3.4.3. Antioxidant Activity

Regarding antioxidant properties, *S. thermophilus* GM4 exhibited a DPPH scavenging activity of 22.7% (Table 4). This indicates a moderate ability to neutralize free radicals. LAB are known to produce various antioxidant compounds, including glutathione, NADH, and antioxidant enzymes, which contribute to their radical scavenging capabilities [72,73]. *Lactobacillus* strains have shown DPPH scavenging activities ranging from approximately 11% to 35% [74]. In contrast, *S. thermophilus* GM4 hydroxyl scavenging activity was comparatively lower (5.70%, Table 4). Hydroxyl radicals are highly reactive and pose significant oxidative stress. While some probiotic strains have demonstrated higher hydroxyl radical scavenging activities (e.g., above 35% and even up to 82%) [75], the modest activity observed for GM4 suggests that its primary antioxidant defense might rely on other mechanisms (e.g., EPS production).

#### 3.4.4. EPS Production During Milk Fermentation

*S. thermophilus* GM4 demonstrated differential EPS yields depending on lactose concentration. In skim milk (lactose content 5%), EPS production remained low (0.13 g/L). However, lactose supplementation (10% *w*/*v*) significantly enhanced EPS yield by approximately 20-fold (2.58 g/L, Table 4), suggesting that carbohydrate availability represents a key limiting factor for EPS biosynthesis in dairy fermentations.

### 3.5. Characterization of EPS from S. thermophilus GM4

#### 3.5.1. Purity and Carbohydrate Composition of EPS

The EPS from *S. thermophilus* GM4 had 51% of carbohydrates and the protein content was 122 mg/g (12%). This EPS was mainly composed by glucose (Glc, 53 mol%) and mannose (Man, 29 mol%), followed by uronic acids (8 mol%), ribose (Rib, 6 mol%), arabinose (Ara, 2 mol%), galactose (Gal, 2 mol%), and traces of rhamnose (Rha) (Table 5). Minor sugars (e.g., Rha) are likely attributable to trace contamination from glycoproteins or other medium components (yeast extract) co-purified during extraction.

The EPS composition aligns with several reports on EPS produced by other *S. thermophilus* strains. For example, the EPS from *S. thermophilus* S-3 was also found to be primarily composed of glucose and mannose, with minor amounts of other sugars such as galactose [76]. Another study on EPS from *S. thermophilus* S-3 demonstrated a composition largely consisting of glucose (59.6%), galactose (21.4%), and mannose (19.0%) [77]. While the exact ratios and presence of minor sugars like ribose, rhamnose, and arabinose can vary between strains, the dominance of glucose and mannose appears to be a recurrent theme for *S. thermophilus* and related LAB. Similar structural configurations have also been observed in EPS from *S. thermophilus* ST538 [7].

**Figure 4:**
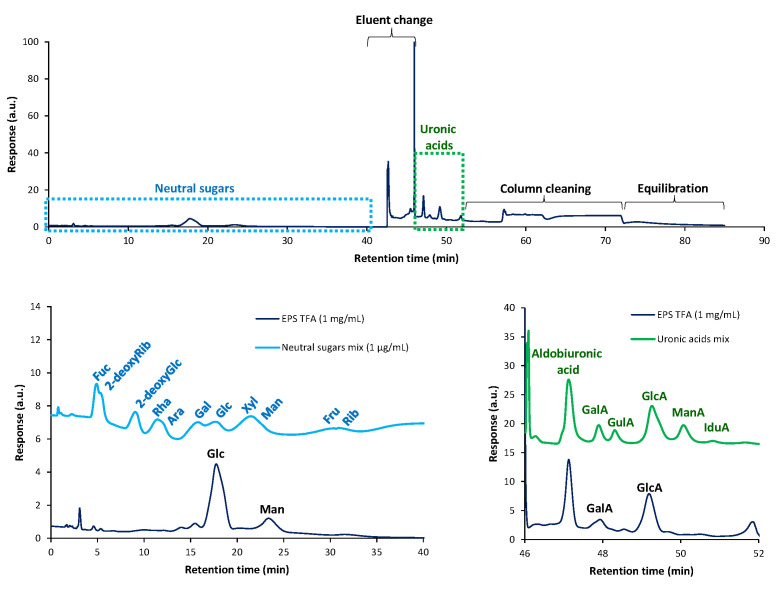
HPAEC-PAD chromatogram of hydrolyzed EPS and comparison with neutral sugars and uronic acids standards.

The analysis of hydrolyzed EPS in HPAEC-PAD confirmed the neutral sugar composition and showed that the main uronic acid of EPS was glucuronic acid followed by galacturonic acid (Figure 4). This profile closely matched those reported in microbial EPS such as *Flavobacterium* (glucuronic and glucose dominant) [78], *Enterobacter* A47 (approx. 29 mol % glucuronic acid) [79], and *Tetraselmis suecica* EPS containing approx. 20–25% glucuronic acid plus minor galacturonic acid (approx. 0.1–3%) [80].

**Figure 5:**
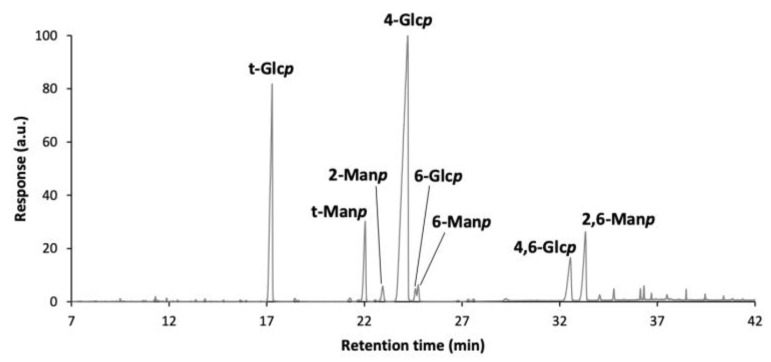
GC-MS chromatogram of partially methylated alditol acetates of EPS from *S. thermophilus* GM4.

To elucidate the glycosidic linkages of EPS, these were methylated and analyzed as partially methylated alditol acetates (PMAAs) by GC-MS (Figure 5). The methodology used only allows to see the glycosidic linkages of neutral sugars, as uronic acids were not carboxyl reduced. The main linkages found in EPS from *S. thermophilus* GM4 (Table 6) were (1→4)-Glc*p* (57.1 mol%), terminally linked Glc*p* (t-Glc*p*, 21.6 mol%), (1→2,6)-Man*p* (7.1 mol%), (1→2)-Man*p* (6.6 mol%), and (1 →4,6)-Glc*p* (5.4 mol%), followed by traces of (1→6)-Man*p* and (1→6)-Glc*p*, (1.1 and 0.9 mol%, respectively).

The presence of lower amounts of deduced linkages for Man, in comparison with previous compositional analysis, could indicate their linkage to uronic acids and formation of aldobiuronic disaccharides, along with methylated EPS hydrolysis, that are lost during PMAA extraction with dichloromethane, before GC-MS analysis. This is in accordance with HPAEC-PAD analysis, where a peak in the elution time of aldobiuronic acid can be seen (Figure 4).

This complex structure of GM4 EPS is consistent with patterns observed in other LAB-derived polysaccharides [8,76].

#### 3.5.2. Molecular Weight Distribution

**Figure 6:**
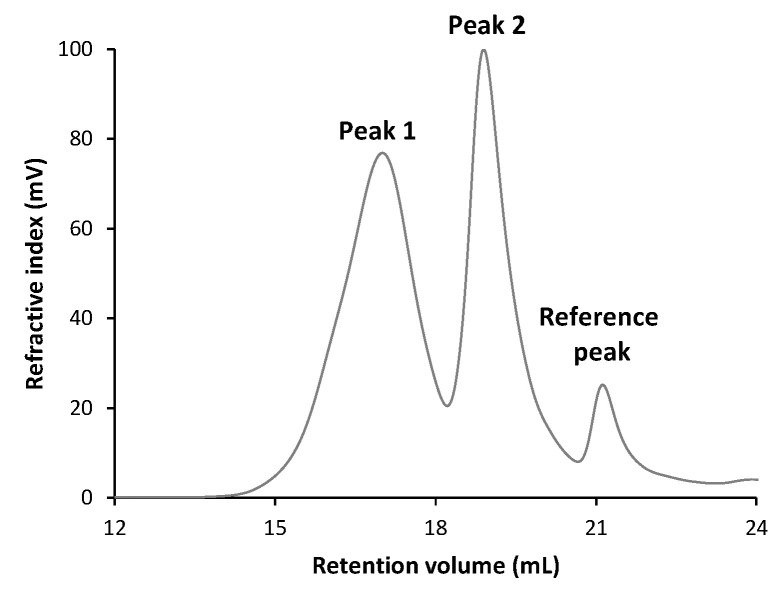
GPC chromatogram of EPS from *S. thermophilus* GM4.

Size-exclusion chromatography (SEC) analysis of the EPS revealed two distinct peaks: Peak 1 at 17.0 min and Peak 2 at 18.9 min, corresponding to two polymer populations of considerably different molecular mass (Figure 6).

The presence of these two populations of polymers gave average values of Mn = 12.0 kDa and Mw = 112 kDa when an overall integration of the chromatogram was performed, revealing a highly polydisperse global distribution (Mw/Mn = 9.27, Table 7). The analysis of each peak showed that Peak 1, associated with a higher molar mass fraction, had average values of Mn = 95.1 kDa and Mw = 189 kDa, while Peak 2’s were Mn = 5.6 kDa and Mw = 10.1 kDa. The dispersity within each peak (Mw/Mn = 1.99 and 1.80, respectively) suggests broad distributions, indicating that each population presents a considerable variation in chain size.

This heterogeneity aligns with reports for other LAB EPS: *Limosilactobacillus reuteri* FW2 produces a bimodal levan (4.6 × 10^6^ Da and 1.2 × 10^4^ Da fractions) [81], while *Lactiplantibacillus plantarum* EPS exhibits similar size polymorphism under different carbon sources [82]. Such multimodal distributions appear characteristic of microbial EPS, likely reflecting biological synthesis mechanisms that generate diverse polymer lengths.

The functional consequences of this molecular diversity are potentially significant: higher-MW fractions may contribute to viscosity, film-forming ability, and immunomodulatory action, while lower-MW fractions could influence solubility, diffusivity, or prebiotic activity. Previous reports link EPS Mw with bioactivity such as antioxidant activity, immune stimulation, and rheological behavior [83].

#### 3.5.3. Dextranase Resistance

The EPS from GM4 exhibited high dextranase resistance (70.3%, Table 4). This level of resistance is similar to that found in EPS from *L. reuteri* [84] and *Leuconostoc citreum* [32], all of which demonstrate high resistance due to specific 4- and 6-linked sugar residues and high molecular weight. While direct studies on dextranase resistance of *S. thermophilus* EPS are limited, comparative data from other LAB species suggest a significant variability in dextranase resistance. For instance, a dextran-type EPS produced by the *Leuconostoc mesenteroides* strain demonstrated only 52% resistance to dextranase degradation [85].

#### 3.5.4. Antioxidant Activity

The EPS from *S. thermophilus* GM4 demonstrated substantial antioxidant capacity, particularly in DPPH radical scavenging, reaching 52.46% at 3 mg/mL (Table 8). This observation aligns with previous reports on LAB-derived EPS, including *S. thermophilus* strains showing 55.83% DPPH scavenging at 1 mg/mL [86]. While the hydroxyl radical scavenging activity was more modest (12.86% at 3 mg/mL), it remains comparable to values reported for other bacterial EPS [71]. This difference in scavenging efficiency between radical types reflects the varying reactivity of these oxygen species and the specific antioxidant mechanisms involved.

The antioxidant properties of the EPS produced by the GM4 strain are likely the result of the structural features of its polymers, particularly the presence of electron-donating functional groups such as hydroxyl and carboxyl groups, which facilitate free radical neutralization [87]. Furthermore, the consistent dose-dependent response observed in both assays underscores the EPS’s potential as a natural antioxidant for food or biomedical applications.

## 4. Conclusions

This study comprehensively characterizes four EPS-producing *S. thermophilus* strains (GM1–GM4) isolated from goat milk, with GM4 emerging as a particularly promising candidate for functional dairy applications. All isolates exhibited efficient lactose fermentation, broad sugar utilization, and desirable enzymatic profiles (notably aminopeptidase and acid phosphatase activity) while lacking harmful enzymes and virulence traits. GM4 was prioritized for further study due to its absence of antibiotic resistance, confirmed safety profile (no hemolytic, DNase, or gelatinase activity), and probiotic potential—demonstrated by gastrointestinal stress tolerance (pH 2.5, bile salts), moderate antimicrobial activity, strong mucin adhesion (69.1%), and pathogen co-aggregation. Technologically, GM4 showed exceptional β-galactosidase activity (56,158.95 ± 1450.28 units) and a versatile carbohydrate fermentation profile. Its EPS, a high-molecular-weight heteropolysaccharide rich in glucose, mannose, and uronic acid with a branched structure, exhibited notable antioxidant capacity (52.46% DPPH scavenging) and dextranase resistance, underscoring its stability and bioactivity. Collectively, these attributes position *S. thermophilus* GM4 as a multifunctional strain capable of enhancing dairy products through simultaneous technological (texture, lactose reduction) and functional (probiotic, antioxidant) benefits, meriting further development for food applications.

## Figures and Tables

**Table 1:** Acidification (ΔpH) in milk after 6 and 24 h; proteolytic and lipolytic activities of *S. thermophilus* strains.

*S. thermophilus* Strains	Acidification		Proteolytic Activity	Lipolytic Activity
	ΔpH 6 h	ΔpH 24 h		
GM1	0.37 ± 0.05 ^a^	1.64 ± 0.04 ^a^	−	−
GM2	0.05 ± 0.01 ^b^	1.32 ± 0.14 ^b^	−	−
GM3	0.05 ± 0.01 ^b^	1.55 ± 0.06 ^a^	+	−
GM4	0.11 ± 0.01 ^b^	1.63 ± 0.09 ^a^	+	−

Different letters as superscripts in the same column indicate a significant difference (*p* < 0.05). Positive (+) and negative (−) results.

**Table 2:** Safety assessments of *S. thermophilus* strains, focusing on virulence-associated enzyme production, antibiotic resistance, and virulence genes.

Strains	Hemolysis	DNase	Gelatinase	Antibiotic Resistance *	Virulence Genes ^†^
GM1	γ	-	-	OXA, VAN, KAN	-
GM2	γ	-	-	OXA, VAN, KAN, GEN	-
GM3	γ	-	-	OXA, TET, STR, ERY	-
GM4	γ	-	-	Sensitive to all	-

* OXA: oxacillin; VAN: vancomycin; KAN: kanamycin; TET: tetracycline; STR: streptomycin, GEN: gentamicin, ERY: Erythromycin. † Virulence genes tested: *gelE*, *hyl*, *asa1*, *esp*, *cylA*, *efaA*, *ace*, *vanA*, *vanB*, *hdc1*, *hdc2*, *tdc*, and *odc*.

**Table 3:** Auto-aggregation (%) and co-aggregation (%) of GM4 with pathogenic bacteria (*E. coli* ATCC 25922, *L. monocytogenes* ATCC 13932, *Staph. aureus* ATCC 25923, and *S. typhimurium* ATCC14028). Results are the average (n = 3) ± SEM.

Auto-aggregation (%)	46.8 ± 0.9
Co-aggregation (%)	
*Escherichia coli* ATCC 25922	36.0 ± 0.2
*Listeria monocytogenes* ATCC 13932	31.4 ± 0.3
*Staphylococcus aureus* ATCC 25923	30.9 ± 0.3
*Salmonella enterica* serovar Typhimurium ATCC14028	26.4 ± 0.2

**Table 4:** Functional characterization of the *S. thermophilus* GM4 strain: *β*-galactosidase activity, cholesterol-lowering ability, antioxidant activity (DPPH and hydroxyl scavenging activities), EPS yield after fermentation (48 h) in skim milk and skim milk supplemented with 10% lactose (Milk + Lac), and dextranase resistance of purified EPS. Results are the average (n = 3) ± SEM.

*β*-galactosidase activity (10^3^ Miller units)	56.2 ± 2.5
Cholesterol biding (%)	26.9 ± 3.3
DPPH scavenging activity (%)	22.7 ± 1.2
Hydroxyl scavenging activity (%)	5.7 ± 0.2
EPS yield milk (g/L)	0.13 ± 0.03
EPS yield milk + Lac (g/L)	2.58 ± 0.05
Dextranase resistance of EPS (%)	70.3 ± 1.2

**Table 5:** Carbohydrate composition of EPS from *S. thermophilus* GM4.

	Sugar Composition (%mol)							Total Sugars (mg/g)
	Rha	Rib	Ara	Man	Gal	Glc	Uronic Acids	
EPS GM4	tr *	6	2	29	2	53	8	509 ± 12

* tr: trace amounts.

**Table 6:** Glycosidic linkage analysis of EPS from *S. thermophilus* GM4.

Deduced Linkages	mol%
t-Glc*p*	21.6
4-Glc*p*	57.1
6-Glc*p*	0.9
4,6-Glc*p*	5.4
**Total Glc** ***p***	**85.0**
2-Man*p*	6.6
6-Man*p*	1.1
2,6-Man*p*	7.1
**Total Man** ***p***	**14.7**

**Table 7:** The weight and number-average molecular weight (Mw and Mn) and polydispersity index (Mw/Mn) of EPS from *S. thermophilus* GM4.

	Total	Peak 1	Peak 2
Mn (g/mol)	12,027	95,102	5614
Mw (g/mol)	111,500	188,959	10,110
Mw/Mn (g/mol)	9.271	1.987	1.801

**Table 8:** Antioxidant activity of EPS produced by *S. thermophilus* GM4.

EPS (mg/mL)	DPPH Scavenging Activity (%)	Hydroxyl Scavenging Activity (%)
1	30.10 ± 2.43	2.41 ± 0.40
2	41.32 ± 0.74	7.26 ± 1.02
3	52.46 ± 0.57	12.86 ± 0.67

## Data Availability

The original contributions presented in this study are included in the article. Further inquiries can be directed to the corresponding author..

## Supplementary Materials

The following supporting information can be downloaded at: https://www.mdpi.com/article/10.3390/foods14173013/s1, Table S1: Primers used for PCR detection of virulence genes [88,89,90].
