# Supplementary material for: Exopolysaccharide (EPS)-Producing Streptococcus thermophilus: Functional and Probiotic Potential
Source: Foods. 2025 Aug 28;14(17):3013. doi: 10.3390/foods14173013 (PMC12428723; doi:10.3390/foods14173013)
Supplement: Supplementary file 1 [file foods-14-03013-s001.zip › foods-3836151-supplementary materials.pdf]

Table S1. Primers used for PCR detection of virulence genes.

Target Gene	Primers Sequences	Fragment Size (bp)	Reference
Gelatinase (*gelE*)	TATGACAATGCTTTTTGGGAT<br>AGATGCACCCGAAATAATATA	213	[1]
Hyaluronidase (*hyl*)	ACAGAAGAGCTGCAGGAAATG<br>GACTGACGTCCAAGTTTCCAA	276	[1]
Aggregation substance (*asa1*)	GCACGCTATTACGAACTATGA<br>TAAGAAAGAACATCACCACGA	375	[1]
Enteroccocal surface protein (*esp*)	AGATTTCATCTTTGATTCTTG<br>AATTGATTCTTTAGCATCTGG	510	[1]
Cytolisin (*cylA*)	ACTCGGGGATTGATAGGC<br>GCTGCTAAAGCTGCGCTT	688	[1]
Endocarditis antigen (*efaA*)	GCCAATTGGGACAGACCTC<br>CGCCTTCTGTTCCTTCTTTGGC	688	[2]
Adhesion of collagen (*ace*)	GAATTGAGCAAAAGTTCAATCG<br>GTCTGTCTTTTCACTTGTTTC	1008	[2]
Vancomycin resistance (*vanA*)	TCTGCAATAGAGATAGCCGC<br>GGAGTAGCTATCCAGCATT	377	[2]
Vancomycin resistance (*vanB*)	GCTCCGCAGCCTGCATGGACA<br>ACGATGCCGCCATCCTCCTGC	529	[2]
Histidine decarboxylase (*hdc1*)	AGATGGTATTGTTTCTTATG<br>AGACCATACACCATAACCTT	367	[3]
Histidine decarboxylase (*hdc2*)	AAYTCNTTYGAYTTYGARAARGARG<br>ATNGGNGANCCDATCATYTTRTGNCC	534	[3]
Tryrosine decarboxylase (*tdc*)	GAYATNATNGGGNATNGGNYTNGAYCARG<br>CCRTARTCNGGNATAGCRAARTCNGTRTG	924	[3]
Ornithine decarboxylase (*odc*)	GTNTTYAAYGCNGAYAARCANTAYTTTGT<br>ATNGARTTNAGTTCRCAYTTYTCNGG	1446	[3]

References

1. Vankerckhoven, V.; Van Autgaerden, T.; Vael, C.; Lammens, C.; Chapelle, S.; Rossi, R.; Jabes, D.; Goossens, H. Development of a multiplex PCR for the detection of *asa1, gelE, cylA, esp,* and *hyl* genes in enterococci and survey for virulence determinants among European hospital isolates of *Enterococcus faecium*. *J. Clin. Microbiol.* **2004**, *42*, 4473–4479.
2. Martín-Platero, A.M.; Valdivia, E.; Maqueda, M.; Martínez-Bueno, M. Characterization and safety evaluation of enterococci isolated from Spanish goats' milk cheeses. *Int. J. Food Microbiol.* **2009**, *132*, 24–32.
3. Rivas, P.; Alonso, J.; Moya, J.; de Gorgolas, M.; Martinell, J.; Fernandez Guerrero, M.L. The impact of hospital-acquired infections on the microbial etiology and prognosis of late-onset prosthetic valve endocarditis. *Chest* **2005**, *128*, 764–771.
